# Effects and Constraints of Optical Filtering on Ambient Light Suppression in LED-Based Underwater Communications

**DOI:** 10.3390/s18113710

**Published:** 2018-10-31

**Authors:** Jan Sticklus, Martin Hieronymi, Peter Adam Hoeher

**Affiliations:** 1 GEOMAR Helmholtz Centre for Ocean Research Kiel, 24148 Kiel, Germany; 2 Institute of Coastal Research, Helmholtz-Zentrum Geesthacht, 21502 Geesthacht, Germany; Martin.Hieronymi@hzg.de; 3 Faculty of Engineering, University of Kiel, 24143 Kiel, Germany; ph@tf.uni-kiel.de

**Keywords:** underwater optical communication, thin film filters, photodetector, ambient light, shot noise

## Abstract

Optical communication promises to be a high-rate supplement for acoustic communication in short-range underwater applications. In the photic zone of oceanic and coastal waters, underwater optical communication systems are exposed by remaining sunlight. This ambient light generates additional noise in photodetectors, thus degrading system performance. This effect can be diminished by the use of optical filters. This paper investigates light field characteristics of different water types and potential interactions with optical underwater communication. A colored glass and different thin film bandpass filters are examined as filter/detector combinations under varying light and water conditions, and their physical constraints are depicted. This is underlined by various spectral measurements as well as optical signal-to-noise ratio calculations. The importance of matching the characteristics of the light emitting diode (LED) light source, the photodetector, and the filter on the ambient conditions using wider angle of incidents is emphasized.

## 1. Introduction

Subsea exploration using advanced technology demands sophisticated underwater communication. Traditionally, the acoustic channel was entirely used, offering long range but limited speed. Attempts have been going on for more than a decade to use the optical underwater channel complementary for communication purposes, promising higher data rates at the expense of lower achievable distances [1,2]. In a variety of scenarios, underwater vehicles such as remotely operated vehicles (ROVs), autonomous underwater vehicles (AUVs), gliders and crawlers are playing a steadily increasing role. Potential applications range from communication between ship hulls, buoys, anchored observatories to inter-vehicle communication between a swarm of autonomous robots in scientific and commercial exploration missions.

Due to cost and small number of available vehicles, particularly for the purpose of field tests, the use of mid-size AUVs with limited depth rating and payload is expected. Hence, these tests and particularly later services will take place in the upper zones of coastal and oceanic waters. In shallow depths the underwater optical communication (UWOC) systems are exposed to ambient sunlight. This is an unavoidable source of disturbance in terms of saturation and noise, and can restrict the serviceability. These undesirable effects can be reduced by integration of matching optical filters to the detectors. In contrast to stationary applications, mobile ones generally require wider field of views (FOVs) for transmitting and receiving. To achieve a hemispherical or even spherical characteristic, it is self-evident to combine segments comprising off-the-shelf parts with limited FOV [3,4,5,6]. Alternatively, an appropriate optical concentrator could be used to extend the FOV of a single detector. However, these are rare for underwater applications and only known for photo multiplier tubes (PMTs) [7].

Because of their narrow beam and high demands with respect to pointing and tracking, lasers and laser diodes (LD) are mostly used as light sources in the special case of stationary setups. They are well known known for their high performance in terms of speed and range and play a big role in visible light communication (VLC) and also in underwater communications [8,9,10,11,12]. On the other hand, light emitting diodes (LEDs) offer significantly lower speed and power density and require more optical bandwidth but provide a wide radiation pattern [13]. Nevertheless, they are considered to be an eligible light source at low cost. Focusing on price and low integration effort, in this study photodiodes are considered rather than silicon photo multipliers (SiPMs) or PMTs, since the strength of the latter ones is limited to low-light applications. The topic of ambient light disturbance in the area of optical communication has been addressed in [14] for the infrared radiation (IR) range, subsequently for VLC in [15,16,17]. Many studies in the field of UWOC make the assumption of total darkness in the depth. To the best knowledge of the authors there are only a few relevant studies of UWOC including solar noise impact in the visible light range [18,19,20] and the ultraviolet (UV) light range [21,22]. In [20] two thin film filters have been tested for the use in an UWOC system due to impairment of sunlight. Thin film filter design has been investigated for multi-color VLC in [23].

This work intends to support UWOC system designers concerning the impact of ambient light with respect to efficient, feasible, and versatile layouts. The remainder of this paper is organized as follows. We present and discuss the key elements of UWOC, beginning with an introduction of the light properties in the medium water under different conditions in Section 2. Related properties of an LED light source and a silicon photodetector are given in Section 3 and Section 4, respectively. Afterwards, in Section 5 different optical filters are described. In Section 6 the performance of diverse filter/detector configurations is examined under the influence of variable ambient light conditions. Finally, concluding remarks are provided in Section 7. Measurement methods are documented in Appendix A.

## 2. The Underwater Light Field

Optical properties of natural waters are very diverse, regional, and temporal along the water column. Besides pure seawater, other water constituents influence the total absorption and scattering properties and hence, the water clarity. These include mainly phytoplankton (algae), suspended matter (debris of plankton or sediments), and colored dissolved organic matter (CDOM). The clarity of natural waters is commonly classified after Jerlov [24], under the assumption of a homogeneous vertical distribution of water constituents within the upper mixed layer of the sea, clear sky, and high solar altitudes. The Jerlov classification scheme differentiates oceanic types (I to III, with subdivision IA and IB) and coastal water types (C1 to C9). In the following, the light field properties of three widespread water types are examined more closely, namely types IB, III, and C3. Jerlov’s classification is based on only one parameter, the irradiance transmittance for surface water. From a comparatively simple depth-profile measurement of downwelling plane irradiance $E_{d}\left(z,\lambda \right)$, one can determine the diffuse attenuation coefficient of downwelling irradiance

(1) $$K_{d}\left(z,\lambda \right)=-\frac{1}{E_{d}\left(z,\lambda \right)}\cdot \frac{dE_{d}\left(z,\lambda \right)}{dz}.$$

The irradiance transmittance T(z,λ) at wavelength λ from the surface to depth *z*, is related to (the assumed depth-constant) attenuation coefficient $K_{d}$ via

(2) $$T\left(z,\lambda \right)=e^{-K_{d}\left(\lambda \right)z}.$$

**Figure 1:**
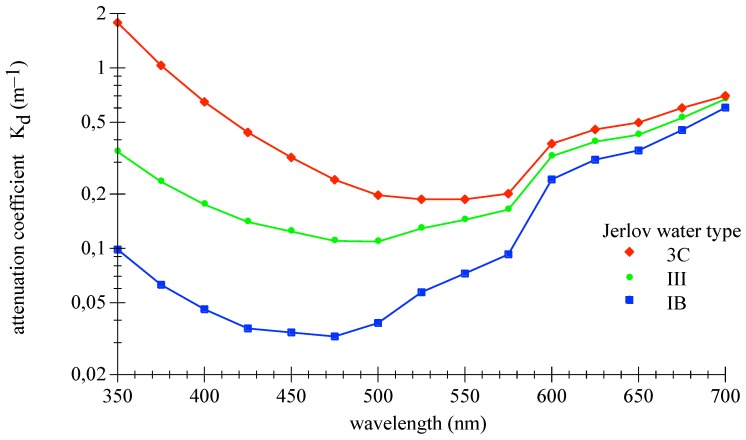
Spectral diffuse attenuation coefficients, $K_{d}$, of downwelling irradiance for three Jerlov water types, replotted from database [25].

$K_{d}$ is an apparent optical property of the water body that varies systematically with wavelength and is rather insensitive to external environmental conditions such as solar zenith angle variations [25]. $K_{d}$ is basically the measure of how sunlight and skylight is attenuated in the water body. Figure 1 shows the spectral downwelling diffuse attenuation coefficients for the selected three water types. The clearest water exhibits a $K_{d}$ minimum in the blue spectral range at 475 nm, whereas the minimum is shifted into the green range for more turbid coastal water.

In contrast to diffuse light, for collimated light in low scattering regimes, the use of the spectral beam attenuation coefficient *c* is appropriate, where *a* is the absorption coefficient and *b* the scattering coefficient. In multiple scattering regimes the use would underestimate ranges since once scattered light is taken account of, a system attenuation coefficient $K_{sys}$ can be more accurate; see [26]. The beam attenuation coefficient *c* can be calculated as follows:

(3) c(λ)=a(λ)+b(λ).

Solonenko and Mobley [25] estimated the fundamental inherent optical properties, i.e., absorption and scattering coefficients of the water constituents, of all Jerlov water types. For our investigations, their derived parameters have been used to run radiative transfer simulations with Hydrolight (version 5.2, Numerical Optics Ltd., Devon, UK); please refer to [27] and to the Appendix A.1. The numerical model computes the in-water radiance distribution as a function of depth and wavelength and provides many other quantities of interest to optical oceanographers.

**Figure 2:**
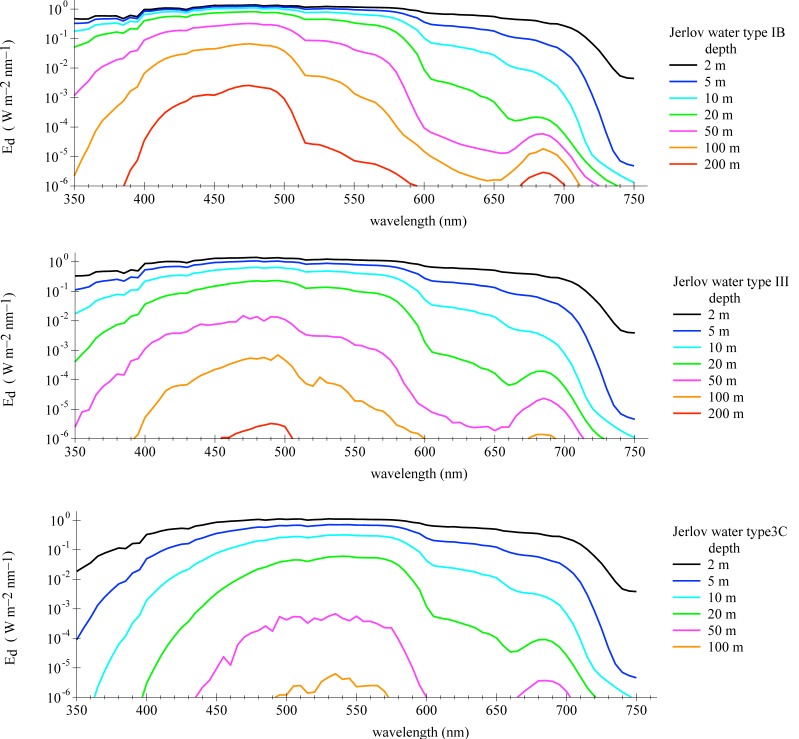
Spectral downwelling Irradiance $E_{d}$ as function of the water depth for three Jerlov water types, under following conditions: sun zenith angle at 30${}^{\circ }$, clear sky, wind speed 5 m/s.

Figure 2 shows the computed decline of spectral downwelling irradiance with water depth for the three water types. In these simulations, the sky is cloud-free with the sun at 30${}^{\circ }$ zenith angle (corresponding to a sun elevation noon at summer in central Europe) and a gentle wind breeze of 5 m/s. The spectral range between 400 and 700 nm is most relevant for phytoplankton; the photosynthetically available radiation (PAR) is the integral over this range. The euphotic depth is defined as water depth, where the PAR downwelling irradiance is 1 % of its surface value. Below this zone there is not sufficient light for plants to carry out photosynthesis (aphotic zone). Based on PAR estimates from the shown $E_{d}$, the euphotic depths are approximately 100, 36, and 22 m for the water types IB, III, and 3C, respectively. These proxy depths would reduce in the case of higher wind speeds, lower sun altitude or more diffuse total insolation. Additional Hydrolight simulations have been carried out with different sun zenith angles (30${}^{\circ }$, 50${}^{\circ }$, and 70${}^{\circ }$) as well as for clear sky and overcast atmospheric conditions.

**Figure 3:**
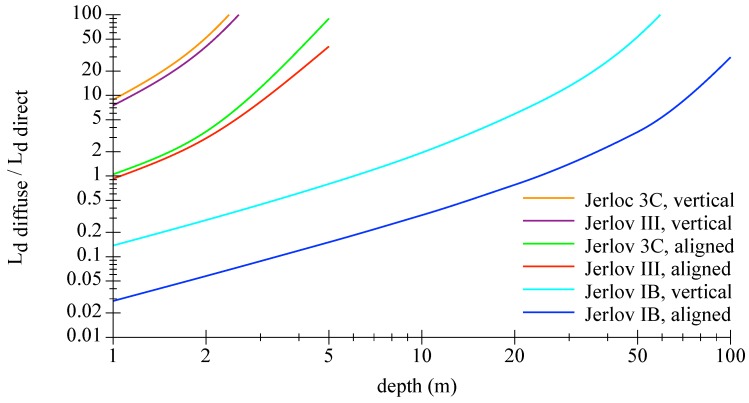
Graphic showing the ratio of the diffuse to the direct radiances as function of the water depth for Jerlov water types IB, III, 3C for a sensor looking vertical upwards or aligned direct to the sun. Data were generated with Hydrolight for a specific wavelength of 450 nm.

The underwater light field and its change with depth is an important factor for further investigations of optical filters. Near the surface, the average downward directed irradiance is generally more than one magnitude higher than the upwelling irradiance. With increasing depth, the shape of the downwelling radiance field gradually transforms from being directed (with radiance maximum from sun zenith direction) to diffuse. Figure 3 shows how the impact of the direct downwelling radiance blurs with depth for the three water types and two viewing directions. The downwelling radiance field is mostly diffuse, i.e., homogeneous from the entire upper half space, in 2, 5, and 20 m for 3C, III, and IB, respectively. Notably, and as a rough guide, an overcast sky reduces the downward irradiance to a quarter, just as the shift of the sun zenith angle from 30 to 70${}^{\circ }$, e.g., noon to evening.

In addition to the time-averaged radiance distribution that Hydrolight computes, we have to deal with extreme radiance fluctuations due to sunlight focusing and defocusing of sea surface waves. At the wind speed under consideration and in the case of clear water, maximum irradiance peaks can exceed the mean irradiance at blue-green wavelengths by a factor of more than 10 near the surface [28,29,30]. The light can fluctuate on time scales as short as milliseconds and distances of less than 1 cm and the intensity of these light fluctuations is higher at longer light wavelengths compared with shorter wavelengths within the visible spectral range [29]. In the presence of larger gravity waves at sea, an enhancement of 50% compared to the mean can still occur at 30 m depth but with decelerated frequency [28]. The intensity and frequency of downward radiance fluctuations is reduced in case of overcast sky just as in case of more turbid water [30]. Thus, radiance fluctuations, mainly from the direction of the direct sun, can be a significant source of disturbances in shallow depths. This fact has to be considered in UWOC operations. Apart from wave-induced light fluctuations and the sensor pointing direct into the sun direction, the sensor orientation facing upwards is generally the worst case for ambient light exposure.

## 3. LED Light Source

An area of least attenuation of light in oceanic waters can be identified at a spectral band of wavelengths in the blue range. For coastal waters this shifts towards green; see [25]. Due to their generally high electro-optical efficiency and speed, blue LEDs are a suitable choice for the use as UWOC transmitters; see [31]. For further investigation, deep blue LEDs in the 450 nm range were chosen. The spectral emission of these LED is roughly Gaussian shaped with a full width at half maximum (FWHM) of typically 20–25 nm. Influencing factors of the LEDs peak wavelength are temperature, current and binning, these can add up to several nanometers in shift; see Table 1. Since this can cause problems in combination with narrow bandwidth optical filters, a careful system design is recommended.

**Figure 4:**
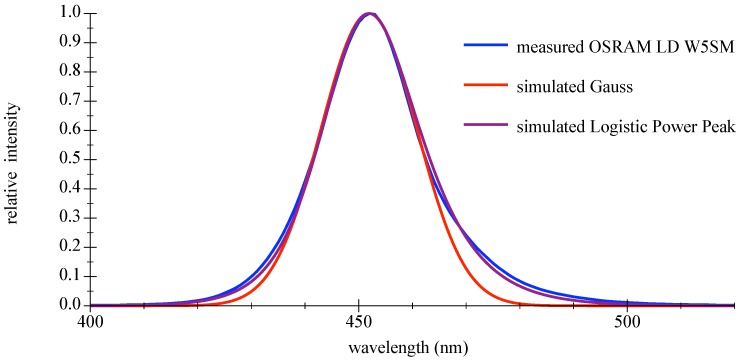
Curves of measured and simulated relative intensity for a deep blue LED.

The use of the LED radiant emission is dependent on the spectral coverage. The device under test (DUT) was a high-power LED LD W5SM (OSRAM Opto Semiconductors GmbH, Regensburg, Germany), driven at $I_{F}=700$ mA measured with a BTS 256 spectrometer (Gigahertz Optic GmbH, Türkenfeld, Germany). The peak center was determined to be 451.9 nm with a FWHM bandwidth of 21 nm. For model functions and parameters please refer to the appendix in Appendix A.2. Graphics in Figure 4 show the measured relative spectral emission of the deep blue LED as DUT compared with the commonly used Gaussian approximation and the better matching logistic power peak model function; please refer to [33]. A comparison is given in Table 2.

The assumed light source for further investigation represents a practically possible single segment of a hemispherical transmitter, containing a cluster of eight LEDs OSRAM LD W5SM combined with a 60${}^{\circ }$ viewing angle reflector. Driving these LEDs with $I_{F}=700$ mA corresponds to a total radiant power of about 6 Watts for the best brightness binning group, and calculations are based on datasheets values; see [32].

## 4. Photodetectors

### 4.1. Types and Characteristics

For intermediate light conditions, large-area silicon-positive intrinsic-negative photodiode (Si PIN PDs) can be an appropriate choice. The higher sensitivity offered from other photodetector types such as SiPMs, PMTs or avalanche photodiodes (APDs) may not be advantageous, since the operating environment in shallower depths is mostly exposed to some ambient light; refer to [19]. Alternative photodetectors for the lower part of the visible spectrum could be in gallium phosphide (GaP) technology, but these specialized products generally offer lower sensitivity and higher capacitance. Pre-assembled Si PIN photodetector-filter combinations are rare on the market and only available for specialized applications. In most instances they are filtered for photo-optical light measurements by reproducing the International Electrotechnical Commission (IEC) curve of human eye color sensitivity, or with flat response over a wide range for radiometric measurements.

**Figure 5:**
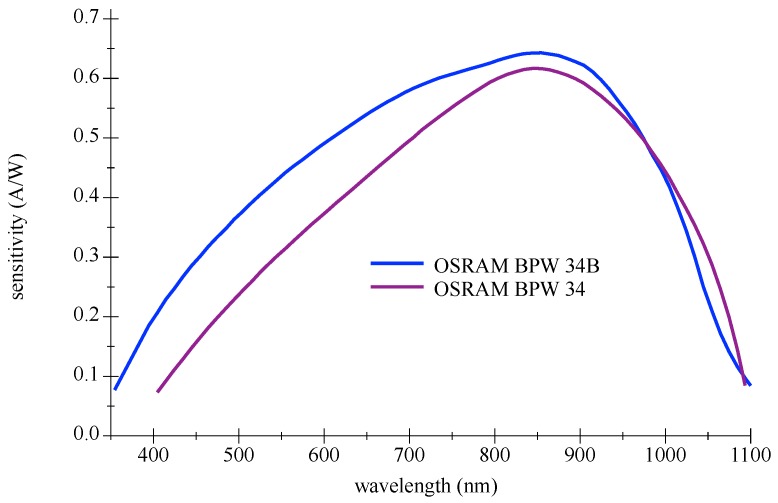
Spectral sensitivity curves for OSRAM BPW 34 and BPW 34B Si PIN Photodetector. Redrawn with values from datasheets [34,35].

**Figure 6:**
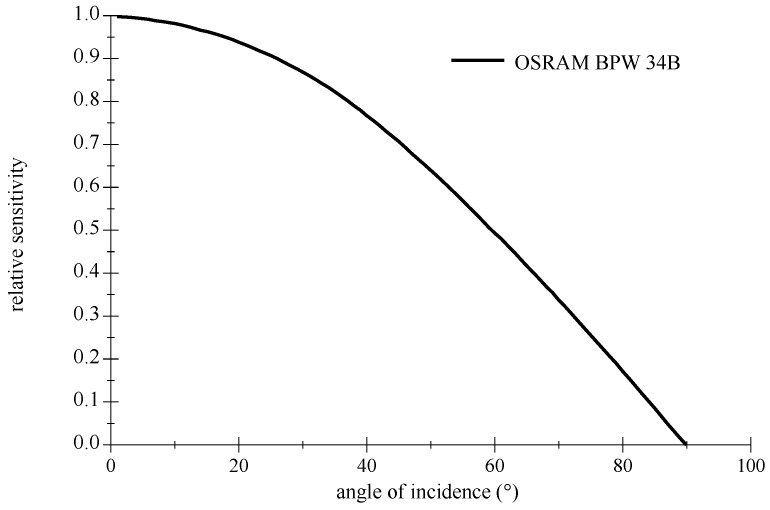
Directional characteristics of an OSRAM BPW 34B Si PIN Photodetector. Redrawn with values from datasheet [34].

A commonly used Si PIN PD is the BPW 34B (OSRAM Opto Semiconductors GmbH, Regensburg, Germany), it is blue enhanced and suitable for the combination to a deep blue LED. Figure 5 shows the difference to a standard non-enhanced BPW 34 detector (OSRAM Opto Semiconductors GmbH, Regensburg, Germany). This planar off-the-shelf type offers a large FOV; please refer to graphics in Figure 6. Furthermore, their relative low capacitance facilitates higher frequencies. As is typical for Si PIN PDs, the maximum of the sensitivity is around 900 nm in wavelength and still significant at 1100 nm.

### 4.2. Amplification

**Figure 7:**
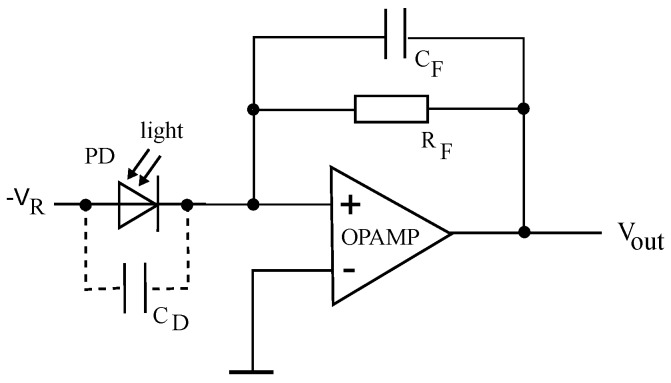
Drawing shows a basic TIA with photodetector. $R_{F}$ and $C_{F}$ are the feedback resistor and capacitor to the operational amplifier. $C_{D}$ is the capacitance of the photodetector, $V_{R}$ the reverse voltage to reduce this capacitance and $V_{out}$ is the output voltage.

The electrical front end of the receiver in UWOC normally consists of the PD itself and a low-noise wideband preamplifier in transimpedance configuration; see Figure 7. Generally, the limiting factor of the bandwidth is the PD capacitance. Another issue is the amplifier saturation due to ambient light, where spectral power density can be orders of magnitude higher as the signal in small water depths. More detailed information about the transimpedance amplifier (TIA) and other circuit configurations can be found in [14,36].

### 4.3. Noise Sources

With respect to the achievable communication speed of a UWOC system, the signal-to-noise ratio (SNR) is the most relevant parameter. The signal power in LED-driven and battery-supplied systems is limited and also attenuated geometrically and exponentially by the water with increasing distance. This implies special attention to the noise, for which several sources and distributors can be identified. The dominant role as sources generally belongs to the Johnson or thermal noise of the feedback resistor $R_{F}$, the preamplifier noise and the shot noise. Shot noise develops if charge carriers cross a potential barrier in p-n junctions and has a random character and is Poisson distributed which shifts to Gaussian for large numbers of events [37]. Shot noise will be generated in the photodetector by its dark current $i_{dark}$, and the currents induced by the ambient and the signals incident light, $i_{amb}$ respectively $i_{sig}$. The corresponding noise equivalent powers (NEPs) can calculated by the following equations [18,38]:

(4) $$NEP_{tR_{F}}=\sqrt{\frac{4\cdot k\cdot T\cdot BW_{en}}{R_{F}\cdot S^{2}}}$$

(5) $$NEP_{i_{namp}}=\frac{i_{namp}\cdot \sqrt{BW_{en}}}{S}$$

(6) $$NEP_{i_{dark}}=\sqrt{\frac{2\cdot q\cdot i_{dark}\cdot BW_{en}}{S^{2}}}$$

(7) $$NEP_{i_{amb}}=\sqrt{\frac{2\cdot q\cdot i_{amb}\cdot BW_{en}}{S^{2}}}=\frac{\sqrt{2\cdot q\cdot S\cdot P_{amb}\cdot BW_{en}}}{S}$$

(8) $$NEP_{i_{sig}}=\sqrt{\frac{2\cdot q\cdot i_{sig}\cdot BW_{en}}{S^{2}}}=\frac{\sqrt{2\cdot q\cdot S\cdot P_{sig}\cdot BW_{en}}}{S}$$

(9) $$NEP_{total}=\sqrt{NEP_{tR_{F}}^{2}+NEP_{i_{namp}}^{2}+NEP_{i_{dark}}^{2}+NEP_{i_{amb}}^{2}+NEP_{i_{sig}}^{2}}$$

where *k* is the Boltzmann constant, *T* is the temperature, *q* is the electronic charge, $BW_{en}$ is the effective noise bandwidth, *S* is the spectral sensitivity, $P_{amb}$ is the signal power, $P_{sig}$ is the ambient light power and $i_{namp}$ is the amplifier current noise density. In the case of shallow water UWOC the most likely dominant noise contributor is shot noise generated by ambient light, analog to typical applications in outdoor visible light communications and wireless infrared communications [14]. A reduction of this shot noise can be achieved by optical bandpass filtering in front of the PD; electronic high pass filtering can only remove the direct current (DC) components but has no effect on the PD shot noise nor the resistors thermal noise.

## 5. Filters

### 5.1. Terminology for Optical Bandpass Filters

**Figure 8:**
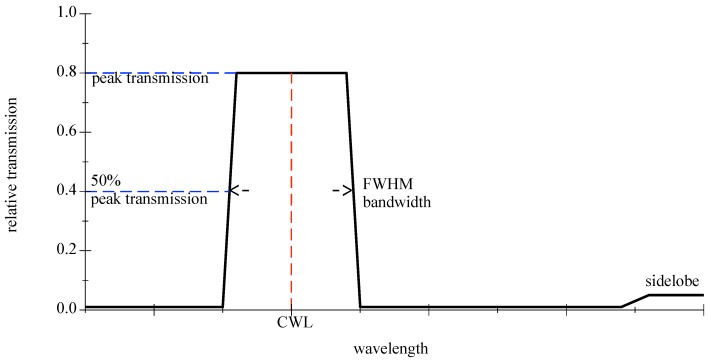
Graphic is showing an idealized bandpass filter.

Optical bandpass filters are generally identified by their center wavelength (CWL) and their bandwidth in the passing range at 50% of the peak transmission, denoted as FWHM, see Figure 8. For further information please refer to [39]. Depending on the type, real bandpasses can show ripples in the distribution, smooth rounded slopes, or sidelobes.

### 5.2. Colored Glass Filter

**Figure 9:**
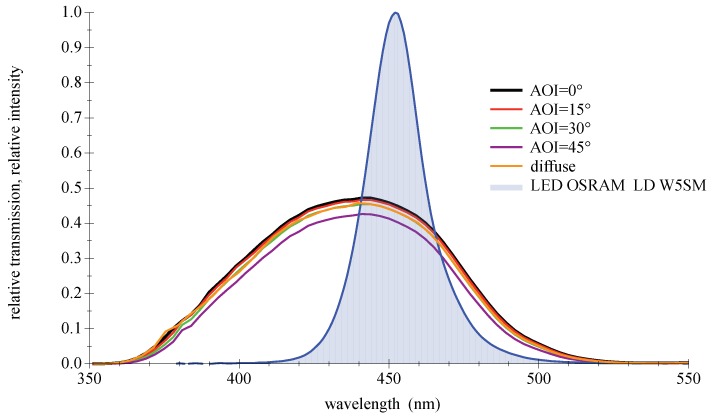
Measured spectral transmission curves for a colored glass bandpass filter Hoya B440 with a directed light source at different AOIs and with a diffuse light source. The measured relative intensity distribution of a deep blue LED OSRAM LD W5SM is also given as filled curve to illustrate the overlap. For further information, please refer to the appendix in Appendix A.3.

Optical filters made of colored glass appear to be simple and low priced. The main effect of these filter types is a more or less selective absorption in a certain wavelength range [40]. As bandpass filters at visible wavelengths, the filters show relatively large transmission losses and weak slopes. Further disadvantages can be fluorescent effects and side lobes. Generally, these filters are available off the shelf in different thicknesses of moderate choice, and not individually made due to the casting production in huge batches. A market survey for bandpass filters matching the deep blue LED center wavelength of 450 nm provides only a few results. Many products are only compatible with each other, limiting the number of discrete types. Since the most suitable filter BG 28 from the manufacturer Schott is discontinued, the B440 type produced by Hoya was chosen for further investigation; please refer to Figure 9 and [41]. An appreciated feature is the relatively low dependence to the AOI, due to a slowly increasing effective thickness at rising angles from the perpendicular of the incident directed light. The characteristic of a particular glass filter can be changed by the material thickness. Increasing it will reduce the transmission and narrow the width of the bandpass. Production related to the glass batches and the thickness may vary slightly. The quality of the filter’s transmission can generally be influenced by the light structure, such as diffuse or directed from a point source. The colored glass filter shows a certain robustness against this alteration. Measurements confirmed only a minor deviation of the curve for diffuse conditions compared to small AOIs.

### 5.3. Thin Film Filter

Thin film filters, also named dichroic or interference filters, are made of many stacked thin layers of alternating high- and low-index material evaporated on a substrate such as glass. Depending on the wavelength, light interferes differently in these layers; it is transmitted or reflected. Generally, these filters are available in a large number of variations, and as individually designed types, but they are typically cost intensive.

**Figure 10:**
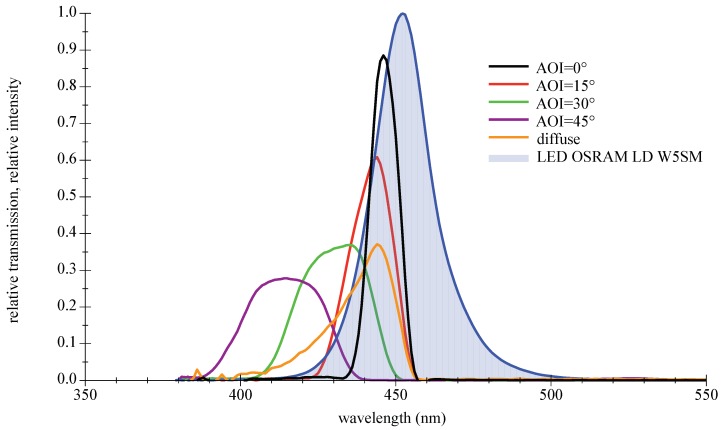
Measured spectral transmission curves for a thin film bandpass filter Thorlabs FBH450-10 with a directed light source at different AOIs and with a diffuse light source. The measured relative intensity distribution of a deep blue LED OSRAM LD W5SM is also given as filled curve to illustrate the overlap. For further information please refer to the appendix in Appendix A.3.

**Figure 11:**
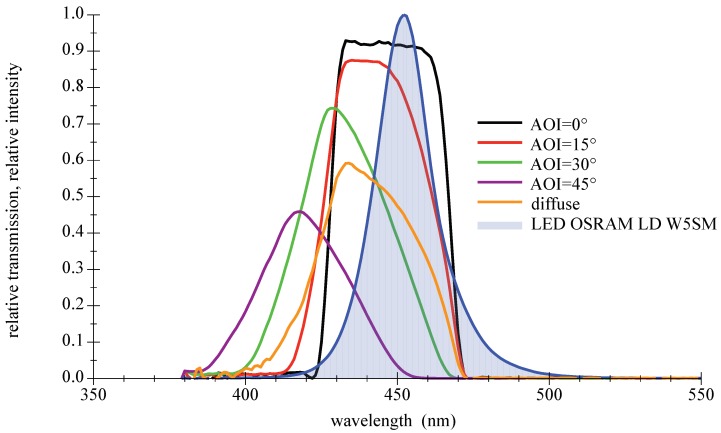
Measured spectral transmission curves for a thin film bandpass filter Thorlabs FB450-40 with a directed light source at different AOIs and with a diffuse light source. The measured relative intensity distribution of a deep blue LED OSRAM LD W5SM is also given as filled curve to illustrate the overlap. For further information please refer to the appendix in Appendix A.3.

**Figure 12:**
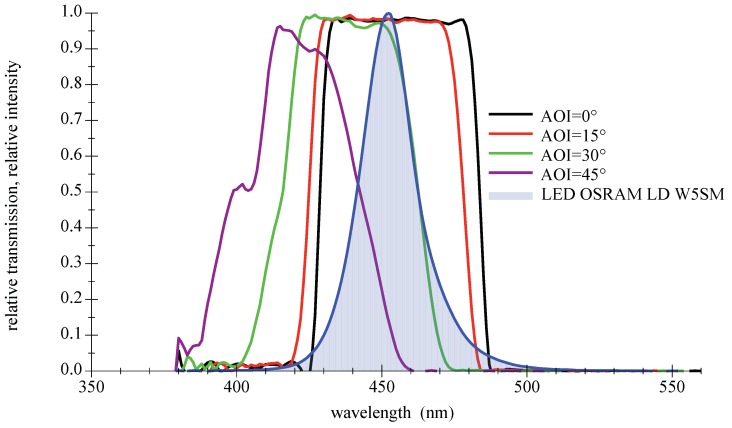
Measured spectral transmission curves for a thin film bandpass filter Semrock 457-50 with a directed light source at different AOIs. The measured relative intensity distribution of a deep blue LED OSRAM LD W5SM is also given as filled curve to illustrate the overlap. For further information please refer to the appendix in Appendix A.3.

Their main advantages are high transmission values and steep slopes [42]. The disadvantage as bandpass filter is the AOI-dependent spectral shift of the transmission zone, which leads to a disintegration of the good characteristics at larger AOIs. Furthermore, thin film filters show an increasing sensitivity with rising AOIs to the polarization direction of light. This only applies to light sources such as lasers or LDs, not LEDs. A selection of filters with different properties suitable for deep blue LEDs are available off the shelf. Three types will be presented and examined in the remainder of this paper: narrow 10 nm bandpass and a wider low cost 40 nm type, both selected to encounter the center wavelength of the LED spectrum; see Figure 10 and Figure 11. The third filter is a high-quality 50 nm bandpass a few nanometers out of alignment towards higher wavelength to compensate for the AOI-related spectral shift in Figure 12. For further information on filter data please refer to [43,44,45].

**Figure 13:**
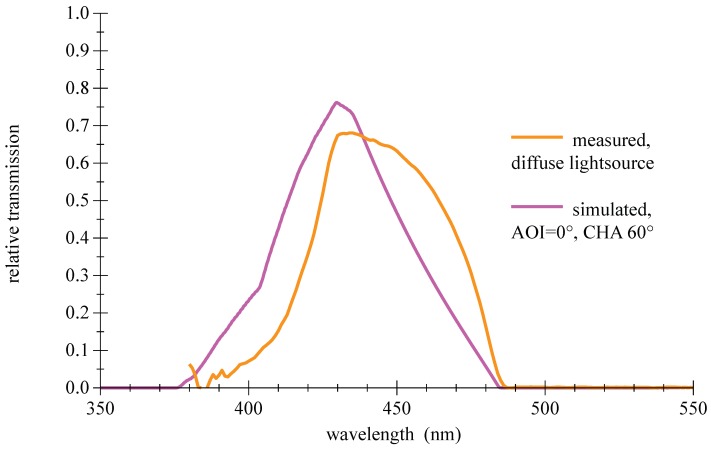
Measured spectral transmission curve for a thin film bandpass filter Semrock 457-50 with diffuse light source and simulated curve for a wide-open cone light source . For further information please refer to the appendix in Appendix A.3.

**Figure 14:**
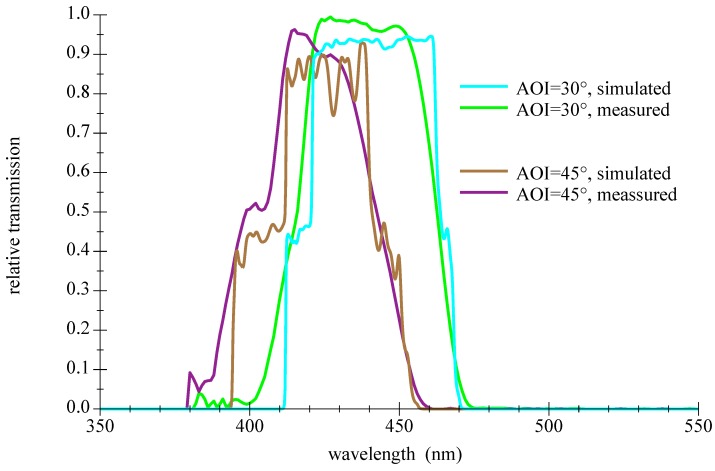
Comparison of measured and simulated spectral transmission curves for a thin film bandpass filter Semrock 457-50 for large AOI. For further information please refer to the appendix in Appendix A.3.

Another drawback of thin film filters is a certain sensitivity to the structure of the incident light; the steep slopes and the transmission are degrading in diffuse light as shown in Figure 13. A comparison of measured and simulated values for demanding conditions of large AOI is given in Figure 14.

### 5.4. Selection and Setup of Components

**Figure 15:**
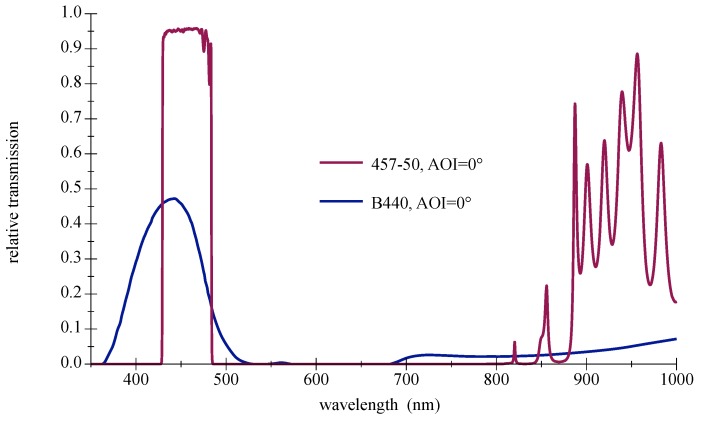
Transmission curves of a colored glass bandpass filter Hoya B440 and a thin film bandpass filter Semrock 457-50. Plot based on manufacturers data [41,45].

To approach the desired goal of a large FOV for single-filtered receiving elements, many parameters need to be considered. Starting at the environmental conditions such as place, water type, operating depth, wavelength of least attenuation, spatial orientation, and ontinued with the light source properties such as LED CWL, LED type, LED operating point, binning, intensity curve. Followed by the filter attributes: FWHM, filter type, CWL, AOI range, coverage of radiant emission, filter quality, tolerances and possible sidelobes; see Figure 15. These sidelobes can be critical, when positioned spectrally inside the sensitivity curve of the detector and the ambient light distribution range; see Figure 5. Proceeding with the photodetector and its spectral sensitivity and directional characteristics, and finally with the choice of the underwater housing technology, this matching process may need some iterations, since many dependencies, including mutual ones, exist.

Anti-reflection coatings can help to reduce losses in transitions to air and make fragile filter surfaces more mechanically robust and resistant. Further optimization can be done by using index matching gel to bond the planar PD to the filter. To set up an optical system for underwater operations it is common to use pressure housings with flat- or dome-ports made of glass or plastics [46], as alternative components can be cast in optical transparent materials such as silicone, polyurethane, or epoxy, depending on the material either in pressure-resistant or pressure-neutral methods of construction [31]. Common to all is to avoid the number and the order of transitions of the optical density, since they may generally influence the transmission and the FOV.

## 6. Signal-to-Noise Evaluation

**Figure 16:**
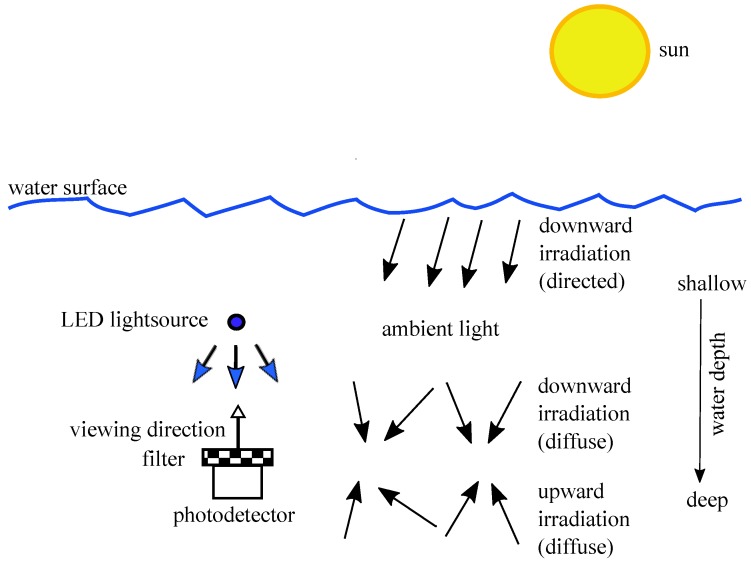
Picture is showing the general scenario and illustrates the environmental circumstances.

The filter implemented in front of a PD should pass the signal from the LED light source and block the ambient light as well as possible. It is unavoidable, that the filter also passes the part of the ambient light spectrum which is within the bandpass; as an unwanted component, this represents optical noise. In Figure 16 the situation underwater is presented for the case of a straight upward-looking sensor.

The optical SNR is defined in Equation (10) and more specifically in Equation (11) with the power of the LED as signal $P_{sigLED}$ and the power of the ambient light as noise $P_{ambLight}$. The spectral measurements deliver irradiation values *E* in Watts per area. For relative calculations, the transition from *E* to the power *P* in Watts is correct, as long the reference area is the same and constant, as for the PD in this case.

(10) $$OSNR=\frac{P_{opt.Signal}}{P_{opt.Noise}}$$

(11) $$OSNR=\frac{P_{sigLED}}{P_{ambLight}}$$

To examine the effectivity of a filter, the change in the optical signal-to-noise ratio (OSNR) is sufficient, and absolute values are not required. This can be calculated as expressed in Equation (12), where $E_{LED}$ is the relative irradiance of the LED and $T_{Filterdir}$ the relative transmittance of the filter for directed light. In the lower part of the fraction, $E_{d}$ is the irradiance of the ambient light, which is in most cases diffuse, and therefore the transmittance of the filter for diffuse light $T_{Filterdiff}$ is used. To achieve values in the more practical unit decibel Equation (13) can be used.

(12) $$\Delta OSNR=\frac{\frac{\int_{350nm}^{750nm}E_{LED}\left(\lambda \right)\cdot T_{Filterdir}\left(\lambda \right)\;d\lambda }{\int_{350nm}^{750nm}E_{LED}\left(\lambda \right)\;d\lambda }}{\frac{\int_{350nm}^{750nm}E_{d}\left(\lambda \right)\cdot T_{Filterdiff}\left(\lambda \right)\;d\lambda }{\int_{350nm}^{750nm}E_{d}\left(\lambda \right)\;d\lambda }}$$

(13) $$\Delta OSNR\;in\;dB=10\cdot log\left(\frac{\int_{350nm}^{750nm}E_{LED}\left(\lambda \right)\cdot T_{Filterdir}\left(\lambda \right)\;d\lambda }{\int_{350nm}^{750nm}E_{LED}\left(\lambda \right)\;d\lambda }\right)-10\cdot log\left(\frac{\int_{350nm}^{750nm}E_{d}\left(\lambda \right)\cdot T_{Filterdiff}\left(\lambda \right)\;d\lambda }{\int_{350nm}^{750nm}E_{d}\left(\lambda \right)\;d\lambda }\right)$$

**Figure 17:**
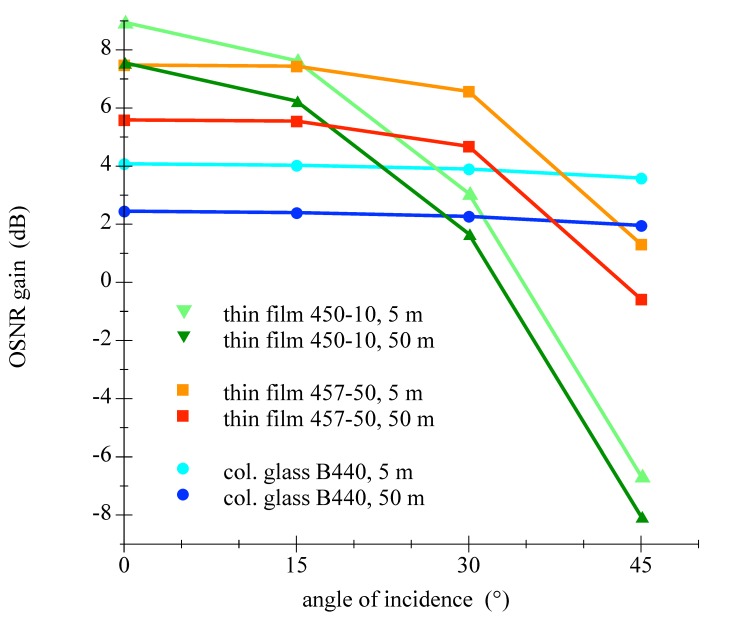
Graphics is showing the change in OSNR as function of the AOI for three different filters at two water depths in water type III (intermediate clear, coastal ocean).

**Figure 18:**
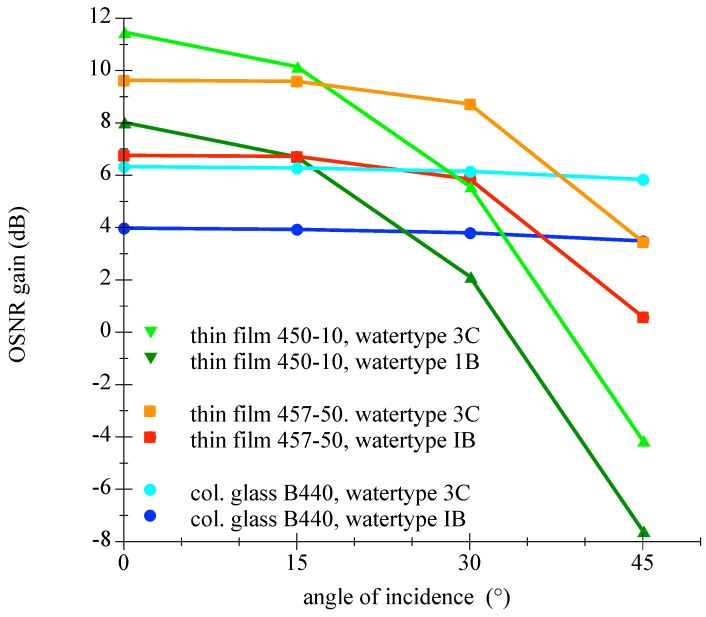
Graphics is showing the change in OSNR as function of the AOI for three different filters at 5 m depth in water types IB (clear ocean) and 3C (coastal water).

Please find further information regarding the calculations in the appendix in Appendix A.4. The upper term of the main fraction in Equation (12) represents the relative passed irradiation of the LED through the filter. Values for four examined different filters with varying AOI are given in Table 3. The lower term means the relative ambient light attenuation by the filter. Since the ambient light spectral composition is a function of the depth and the water type, this is considered in the scope of Table 4. By taking values from Table 3 and Table 4 and using Equation (13), the improvement in OSNR can be calculated for a specific filter, at a certain depth of a given water type for a desired angle of incidence. This has been performed for many cases and the results for varying water depth and is presented in Figure 17 and the outcome for diverse water types is illustrated in Figure 18.

The analysis of the Table 3 and Table 4 and Figure 17 and Figure 18 shows that the Semrock 457-50 thin film bandpass filter would be the best choice of tested components. The attenuation of the LEDs signal is low with simultaneous tolerance of larger AOIs. This result would allow energy efficient applications with 60${}^{\circ }$ FOV with comparatively high improvement of the OSNR compared to a colored glass filter.

## 7. Conclusions

The application of different types of optical filters for underwater communication purposes is examined in this paper. The challenging environment of light conditions underwater as well as the LED as light source are taken in consideration. The photodetector and noise sources are introduced, and several spectral measurements were performed to investigate the transmission as function of the angle of incidence as well as to depict differences between measurements and simulations. The fundamentals for the filter selection are described. The importance of matching components has been emphasized and the filter capability in terms of the optical SNR is investigated. It is shown that a well-selected thin film filter is able to suppress a significant portion of the ambient light and offers robustness to angular dependence and varying water properties. The results of this work can support UWOC system designers in adapting equipment to the large number of influencing factors. Further research on particular adaption to water types, filter optimization procedures, the consideration of LDs as light source and the transition from optical SNR to electronic noise are suggested in future work. 

## Figures and Tables

**Table 1:** Peak wavelength influence factors for a deep blue high-power LED OSRAM LD W5SM. Values calculated based on datasheet [32].

Influence Factor			
temperature gradient	+0.033	nm/K	
current gradient	−0.002	nm/mA	(valid for $I_{F}$ = 500 to 1000 mA)
binning group width	4	nm	(3 groups available)

**Table 2:** Calculated values for spectral coverage and resulting bandwidth at different parts of mean intensities of the measured relative spectral emission and two approximations.

Part from Mean	Measured Curve	Gaussian Approx.	Logistic Power Peak Approx.
full	100%	100%	100%
	150 nm	100 nm	120 nm
0.05	96.6%	98.5%	97.3%
	59 nm	43.6 nm	55.2 nm
0.1	93.6%	96.9%	94.6%
	48.5 nm	38.4 nm	45.6 nm
0.2	87.4%	92.7%	88.9%
	37.5 nm	32 nm	36 nm
0.5	66.1%	76.1%	66.0%
(FWHM)	21 nm	21 nm	20 nm

**Table 3:** Calculated rates of passed radiated power for different LED-filter combinations at various AOIs, based on measured LED spectra and filter transmission curves. These values are equal to signal power loss caused by the optical filter.

AOI	Colored Glass	Thin Film	Thin Film	Thin Film
	Hoya	Thorlabs	Thorlabs	Semrock
	B440	FBH450-10	FB450-40	457-50
0${}^{\circ }$	42.0%	29.1%	77.8%	93.6%
	−3.77 dB	−5.36 dB	−1.09 dB	−0.29 dB
15${}^{\circ }$	41.5%	21.5%	64.1%	92.7%
	−3.82 dB	−6.68 dB	−1.93 dB	−0.33 dB
30${}^{\circ }$	40.3%	7.5%	31.7%	75.4%
	−3.95 dB	−11.25 dB	−4.99 dB	−1.20 dB
45${}^{\circ }$	37.5%	0.8%	6.0%	22.6%
	−4.26 dB	−20.97 dB	−12.22 dB	−6.46 dB

**Table 4:** Calculated ambient light suppression for the four different filters in three exemplary water types at varying depths, up to meaningful remaining light values. Generally calculated with filter characteristics for diffuse light, with the exception for very shallow water of 2 to 5 m in the clear water type IB. Here the characteristics of the dominating directed light were used for thin film filter (AOI = 15${}^{\circ }$); please refer to Figure 3. Values for the thin film filters in water type IB at a depth of 10 m were not calculated, since the filter characteristic either for diffuse or directed light were applicable.

**Water Type IB**	**Depth**						
**Filter**	**2 m**	**5 m**	**10 m**	**20 m**	**50 m**	**100 m**	**200 m**
B440	−8.53 dB	−7.75 dB	−7.14 dB	−6.54 dB	−5.74 dB	−5.31 dB	−5.23 dB
FBH450-10	−14.21 dB	−13.38 dB	n.a.	−12.98 dB	−12.11 dB	−11.76 dB	−12.09 dB
FB450-40	−10.05 dB	−8.22 dB	n.a.	−8.55 dB	−7.61 dB	−7.15 dB	−7.29 dB
457-50	−6.87 dB	−7.05 dB	n.a.	−6.40 dB	−5.40 dB	−4.82 dB	−4.64 dB
**water type III**	**Depth**						
**filter**	**2 m**	**5 m**	**10 m**	**20 m**	**50 m**	**100 m**	
B440	−8.47 dB	−7.85 dB	−7.47 dB	−7.28 dB	−6.22 dB	−6.58 dB	
FBH450-10	−14.90 dB	−14.31 dB	−13.99 dB	−13.99 dB	−12.92 dB	−14.57 dB	
FB450-40	−10.62 dB	−9.96 dB	−9.56 dB	−9.45 dB	−8.40 dB	−9.51 dB	
457-50	−8.47 dB	−7.77 dB	−7.31 dB	−7.03 dB	−5.88 dB	−6.38 dB	
**water type 3C**	**Depth**						
**filter**	**2 m**	**5 m**	**10 m**	**20 m**	**50 m**		
B440	−9.51 dB	−10.1 dB	−11.40 dB	−13.70 dB	−13.99 dB	
FBH450-10	−15.89 dB	−16.83 dB	−19.07 dB	−22.79 dB	−24.25 dB	
FB450-40	−11.64 dB	−12.49 dB	−14.73 dB	−19.43 dB	−22.64 dB	
457-50	−9.36 dB	−9.92 dB	−11.56 dB	−14.98 dB	−16.30 dB	

## Abbreviations

The following abbreviations are used in this manuscript:

AOI	Angle of Incidence
APD	Avalanche Photodiode
AUV	Autonomous Underwater Vehicle
CDOM	Colored Dissolved Organic Matter
CHA	Cone Half Angle
CWL	Center Wavelength
DC	Direct Current
DUT	Device Under Test
FOV	Field of View
FWHM	Full Width at Half Maximum
GaP	Gallium Phosphide
IEC	International Electrotechnical Commission
IR	Infrared Radiation
LD	Laser Diode
LED	Light Emitting Diode
NEP	Noise Equivalent Power
OSNR	Optical Signal-to-Noise Ratio
PAR	Photosynthetically Available Radiation
PMT	Photo Multiplier Tube
ROV	Remotely Operated Vehicle
Si PIN PD	Silicon-Positive Intrinsic-Negative Photodiode
SiPM	Silicon Photo Multiplier
SNR	Signal-to-Noise Ratio
TIA	Trans Impedance Amplifier
UV	Ultraviolet
UWOC	Under Water Optical Communication
VLC	Visible Light Communication

## Appendix A.

### Appendix A.1. Hydrolight Simulations

Radiative transfer simulations were carried out using the commercial software HydroLight version 5.2 (Numerical Optics Ltd., Devon, UK) [27]. HydroLight computes radiance distributions and derived quantities such as irradiances and reflectances for natural water bodies. To characterize the ambient light field in water, simulations have been conducted for three different water types, three sun zenith angles (30${}^{\circ }$, 50${}^{\circ }$, and 70${}^{\circ }$) as well as for clear sky and overcast atmospheric conditions. The chosen optical water types orientate towards Jerlov’s classification: two oceanic types, IB and III, and one coastal type, 3C [24]. The corresponding absorption and scattering properties of these Jerlov water types where estimated by [25]; their parameterizations and results were implemented into HydroLight. The spectral range covers 350 to 750 nm in 5 nm steps. Relevant boundary conditions include default characteristics of seawater, volume scattering properties of average particles, and wind speed of 5 m/s.

### Appendix A.2. LED Spectra Modeling

Parameters used for the Gaussian model function are: A = 1, C = 451.9 and W = 21. Parameters used for the Logistic Power Peak model function are: A = 1, S = 1.4, C = 451.9 and W = 5.55. For the corresponding formulas please refer to [33].

### Appendix A.3. Filter Measurements

The transmission measurements were performed on an optical bench in the dark by using a Gigahertz Optic Spectrometer BTS256 within the range from 380 to 750 nm. Values from 350 to 380 nm were taken from the datasheets provided by the filter manufacturers. As point light sources stabilized halogen units were used. For measurement with diffuse light a sheet of Plexiglas LED 0M200SC (Evonik Industries AG, Darmstadt, Germany) was applied in the beam path. Filter simulations were performed with the modeling tool “MyLight” (Semrock Inc., Rochester, NY, USA). To approach a diffuse character best possible a cone half angle (CHA) of 60${}^{\circ }$ was used.

### Appendix A.4. Signal-to-Noise Calculations

For calculations, the narrow band of the LED spectrum is supposed to have a constant attenuation factor over this band, so no distortion of the relative spectrum will take place for different distances. Integral calculus over a wavelength band were performed by summation of discrete elements of 1 nm width.
